# Remembering Jonathan M. Mann in a World Ajar

**DOI:** 10.3201/eid0909.030381

**Published:** 2003-09

**Authors:** Kathleen F. Gensheimer

**Affiliations:** * Maine Department of Human Services, Augusta, Maine, USA

**Keywords:** Epidemiology, human rights, humanitarian

**To the Editor:** September 2003 marks the anniversary of the deaths of Jonathan M. Mann and his wife Mary Lou Clements aboard Swiss Air flight 111, which crashed off the shore of Peggy’s Cove, Nova Scotia, 5 years ago. Although Jonathan and I were both members of the Council of State and Territorial Epidemiologists in the early 1980s, when Jonathan served as state epidemiologist for New Mexico, our paths did not cross until years later in 1990. Jonathan had reluctantly resigned as director, Global AIDS Activities, World Health Organization, to become full professor at Harvard School of Public Health. I had taken a year’s leave of absence from my position in Maine to enroll in Harvard’s Master of Public Health program.

In a talk at the Centers for Disease Control and Prevention, Jonathan once outlined many of his hopes and fears for AIDS activities worldwide. Moved by his pleas for global commitment to the epidemic, I sought out Jonathan at the opening reception for new Harvard students. I shared his dreams for public health activism. We believed in inspiring others to careers in applied public health, so we initiated a brown bag lunch series for students and faculty to share experiences about work in public health (*1*). The common thread throughout these discussions was universal human rights and respect for human dignity.

Jonathan went on to establish the Francis Xavier Bagnoud Center for Health and Human Rights at the Harvard School of Public Health and used his position to promote health as the broad-based core of human values. His lectures on universal human rights centered on the idea that health transcends geographic, political, economic, and cultural barriers. Jonathan drew on his past experiences with the HIV epidemic to argue that the developing world would never achieve economic or political stability unless the health of its people improved. He maintained that, if not addressed, the health problems of the developing world would pose a global threat. “Public health,” he wrote, “too often studies health without intruding upon larger, societal, inescapably laden issues…. If the public health mission is to assure the conditions in which people can achieve the highest attainable state of physical, mental and social well-being, and if these essential conditions predominantly are societal, then public health must work for societal transformation” (*2*).

Jonathan argued that discrimination and other violations of human rights were primary pathologic forces working against the improvement of public health and that if we ignored the plight of those whose rights were violated, we would be less than human ourselves. Jonathan very much admired Eleanor Roosevelt, chair, Declaration of Human Rights Drafting Committee, who on the 10th anniversary of the declaration asked, “Where, after all, do universal human rights begin? In small places, close to home—so close and so small that they cannot be seen on any map of the world. Such are the places where every man, woman and child seeks equal justice, equal opportunity and, equal dignity. Without concerted citizen action to uphold them close to home, we shall look in vain for progress in the larger world” (*3*).

On Jonathan’s desk at Harvard, amidst family photographs, was a framed joker taken from an ordinary deck of cards. When I asked about its significance, he responded that, despite life's challenges, it remains important to smile. So smile we must at the memory of Jonathan and his many accomplishments. Each year, the Council of State and Territorial Epidemiologists remembers by holding a distinguished lecture named in honor of Jonathan M. Mann.

The public health practitioner must respond to the needs of people and yet be sensitive to world politics. In solving difficult issues, the practitioner must understand the interconnection of social values and scientific truths and work collaboratively with the medical community. Moved to the forefront by recent acts of terrorism, public health has achieved recognition as first responder and as integral part of planning for and responding to catastrophic health crises. We cannot promote safety and security if we fail to recognize, and advocate for, people around the globe who do not have access to basic health care, adequate living and working conditions, or education to enlighten their response to life’s challenges. The anniversary of Dr. Mann’s untimely death serves as reminder to the medical and public health communities of the ongoing need to promote universal human rights and to focus energies and resources on a global approach to public health.
